# Pandemic (H1N1) 2009 Influenza Virus Infection in A Survivor Who Has Recovered from Severe H7N9 Virus Infection, China

**DOI:** 10.3389/fmicb.2016.01514

**Published:** 2016-10-04

**Authors:** Shan-Hui Chen, Meng-Na Wu, Yan-Hua Qian, Guang-Yuan Ma, Guo-Lin Wang, Yang Yang, Teng Zhao, Bing Lu, Mai-Juan Ma, Wu-Chun Cao

**Affiliations:** ^1^Wuxi Center for Disease Control and PreventionWuxi, China; ^2^State Key Laboratory of Pathogen and Biosecurity, Beijing Institute of Microbiology and EpidemiologyBeijing, China; ^3^Department of Biostatistics, University of Florida, GainesvilleFL, USA

**Keywords:** pdm09 (H1N1) virus, H7N9 virus, subsequent infection, serological detection, vaccination

## Abstract

We firstly report a patient who presented with severe complications after infection with influenza A(H1N1) pdm2009, more than 1 year after recovery from severe H7N9 virus infections. The population of patients who recovered from severe H7N9 infections might be at a higher risk to suffer severe complications after seasonal influenza infections, and they should be included in the high-risk populations recommended to receive seasonal influenza vaccination.

## Background

Since the emergence of human infections with avian influenza A (H7N9) virus in February 2013, China has experienced four epidemic waves of this virus. Due to its co-circulation with influenza A (H1N1) pdm09 (pH1N1), seasonal influenza A (H3N2 and H1N1), and influenza B viruses in China, human co-infections with the avian H7N9 virus and these human influenza viruses have been reported (Zhu et al., 2013; Li et al., 2014; Zhang et al., 2015). Most H7N9 patients had severe symptoms during hospitalization and their pulmonary functions were seriously damaged, which might have increased their susceptibility to subsequent viral infections including human seasonal influenza viruses after their recovery from the H7N9 infection. Here, we report a case of pH1N1 virus infection who had survived from H7N9 infection about 1 year and 3 months ago.

## Case Report

**FIGURE 1:**
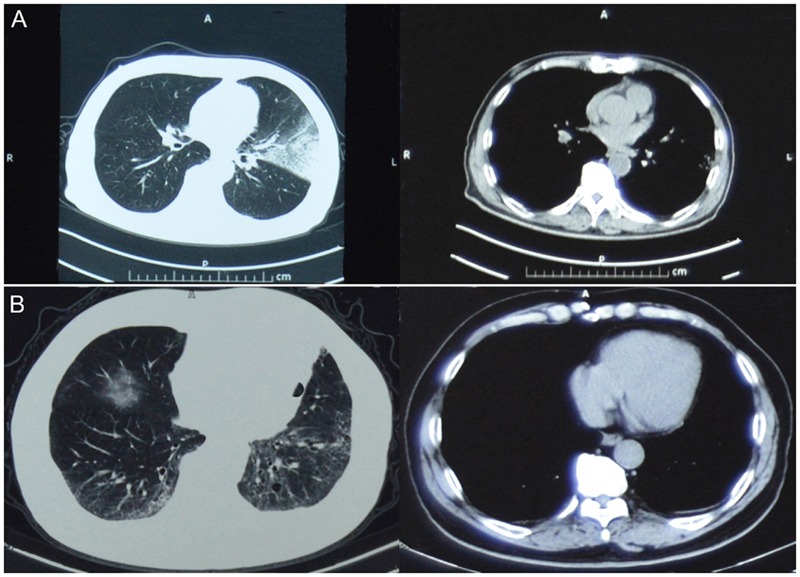
**Computed tomography (CT) images of the patient. (A)** and **(B)** show CT images of the patient infected with H7N9 virus on 25 April 2014 and of the patient infected with pH1N1 virus on 27 December 2015, respectively. Left and right CT images are lung and mediastinal window, respectively.

**FIGURE 2:**
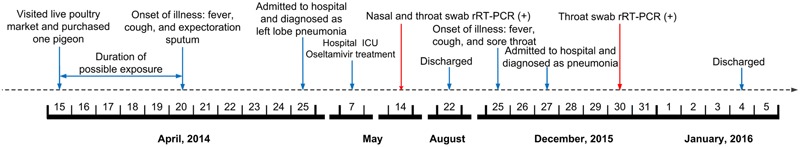
**Timeline of events the patient associated with H7N9 and pH1N1 viruses infections in Wuxi, Jiangsu Province, China.** rRT-PCR, real time reverse transcriptase-polymerase chain reaction; ICU, intensive care unit.

On April 20, 2014, a 66-year-old retired man, who had a history of hypertension for over 20 years, presented influenza-like-illness (ILI) symptoms with fever (38°C), cough, and expectoration sputum. According to the epidemiological investigation, 5 days before symptoms presentation the patient visited a local live poultry market and purchased one pigeon. On April 25, he sought medical care at community clinic when his symptoms were getting worse, where he was diagnosed of left lower lobe pneumonia. Because of his severe conditions, he was transferred to the respiratory department of a city level hospital on the same day and was diagnosed of left pneumonia and chronic bronchitis. The admission assessment of vital signs revealed a temperature of 39°C, a pulse of 101 beats per minute, a blood pressure of 130/80 mmHg, a respirations rate of 22 breaths per minute, a saturation of peripheral oxygen (SpO2) of 99.4%, a pressures of oxygen (PaO2) of 135 mmHg, and a carbon dioxide (PaCO2) of 26.1 mmHg (**Table 1**). Routine laboratory testing revealed a white cell count (WBC) of 3.7 × 10^9^/L with 59.8% neutrophils and 70mg/L C reactive protein (CRP; **Table 1**). Computed tomography (CT) of the chest showed left upper lobe pneumonia, lower lobe interstitial fibrosis, lower lobe predominant emphysema, and mediastinal lymph nodes (**Figure 1A**). On the following day, the patient developed high fever (40.1°C) with cough and expectoration sputum. He was prescribed with ceftriaxone tazobactam and levofloxacin after his hospital admission. When these antibiotics turned out ineffective, he was then treated with imipenem cilastatin, vancomycin, and meropenem. However, due to the poor treatment effectiveness of these medicine and continuous deterioration of the disease condition, he was sent to the intensive care unit on May 7, and given oxygen nasal cannula and mechanical ventilation successively as well as oseltamivir 75 mg twice daily. On May 14, his nasal and throat samples were collected and were tested positive for H7N9 virus using real time reverse transcription PCR (rRT-PCR). After the confirmative diagnosis, he continued to receive oseltamivir 75 mg twice daily with imipenem and moxifloxacin to help alleviate symptoms during May 15–26. On August 22, he recovered from the disease and was discharged from the hospital. **Figure 2** summarizes the timeline of the events mentioned above.

**Table 1:** Clinical characteristics of patient with confirmed H7N9 and pH1N1 infection on hospital admission.

Characteristic	H7N9 infection	pH1N1 infection
Age (years)	66	
Sex	Male	
Occupation	Retired	
Type of exposure	Visited live poultry market and purchased one pigeon	n/a
Underlying medical disorders	Hypertension	Hypertension
Smoking	Yes	No
Influenza vaccination	No	No
Onset of illness	20 April 2014	25 December 2015
Date of hospital admission	25 April 2014	27 December 2015
Date of hospital discharge	22 August 2014	4 January 2016
Signs of illness	Fever, cough, and expectoration sputum	Fever, cough, and sore throat
Temperature (°C)	39.0	38.0
White blood count ( × 10^9^/L)	3.7	11.3
Neutrophils ( × 10^9^/L)	2.49	63%
Lymphocytes ( × 10^9^/L)	0.796	2.39^∗^
Platelets ( × 10^9^/L)	147	195^∗^
C reactive protein (mg/L)	70	9
PaCO_2_ (mm Hg)	26.1	32.5
PaO_2_ (mm Hg)	135	92
Saturation of peripheral oxygen (%)	99.4	n/a
Chest radiography	Pneumonia	Pneumonia
Mechanical ventilation	Yes	No
Oseltamivir treatment	Yes	Yes
Oxygen treatment	Yes	Yes
Outcome	Recovery	Recovery

*n/a, not applicable. ^∗^The data on hospital admission was not applicable and showed the available data on 28 December 2015.*

On December 25, 2015, 1 year and 3 months after his infection with the avian influenza A(H7N9) virus, he presented ILI symptoms again, with fever (38°C), cough, and sore throat. On December 27, he was admitted to the same city level hospital and was diagnosed of severe pneumonia. On admission, the patient has a temperature of 36.3°C, a pulse of 77 beats per minute, a blood pressure of 120/70 mmHg, a respirations rate of 20 breaths per minute, a PaO2 of 92 mmHg, and a PaCO2 of 32.5 mmHg (**Table 1**). Routine blood testing identified a WBC 11.3 × 10^9^/L with 63% neutrophils and 9mg/L CRP (**Table 1**). CT of the chest showed pulmonary interstitial fibrosis with infection (**Figure 1B**). He was prescribed with cefotiam, xiyanping (Chinese medicine for anti-virus infection), doxofylline, and ambroxol as well as given oxygen nasal cannula. On December 29, 2015, the local municipal Center for Disease Control and Prevention was notified of the patient with severe pneumonia. Because of his historical infection with avian influenza A(H7N9) virus, an epidemiological investigation was immediately conducted on December 30, and two throat samples were collected and tested for avian influenza virus subtypes (H7N9, H5N1, and H9N2) and human seasonal influenza viruses (pH1N1 and seasonal H1 and H3) using rRT-PCR. Both samples were positive for influenza A virus (Ct = 32.36) and pH1N1 virus (Ct values are 31.15 for H1 and 32.35 for N1), but negative for H7N9 or other subtypes of influenza viruses. After the confirmation diagnoses of pH1N1 virus infection, his treatment with prescribed drugs continued. On January 4, 2016, he recovered from the disease and was discharged from the hospital (**Figure 2**).

## Hemagglutination Inhibition Assay

The serum hemagglutination inhibition (HAI) antibodies against the H7N9 and pH1N1 were measured as previously described (World Health Organization, 2013). The HAI titers against the H7N9 virus (A/Jiangsu/Wuxi/04/2013) were 1:40 and 1:1280 in serum samples collected on May 15 and June 16 of 2014, respectively. The HAI titer against the pH1N1 virus (A/California/07/2009) was 1:1280 in the convalescent serum samples collected on January 25, 2016. To determine whether the patient had HAI antibodies against the H7N9 virus more than 1 year after his recovery, we tested the convalescent serum for H7N9 virus and found the patient had a low HAI titer of 1:10.

## Discussion

To our knowledge, this is the first report of a survivor of the avian influenza H7N9 infection who were infected with the human influenza pH1N1 virus after his recovery from the disease. A previous study found that H7N9-infected patients had rapidly developed robust antibody responses in 10–14 days after symptom onset (Zhang et al., 2013). Our result also showed a rapidly elicited robust antibody response after the infection, but the antibody response became very weak against H7N9 virus more than 1 year later. Whether the protective antibody response decay substantially after 1 year or so in general need further investigation in more survivors, and there is likely heterogeneity across the population. Several studies have shown that memory T cells and broadly neutralizing antibodies induced by human seasonal influenza viruses may provide some cross-protection against the avian H7N9 virus (van de Sandt et al., 2014; Henry Dunand et al., 2015; Richards et al., 2015). It is yet unclear whether previous exposure to the avian H7N9 virus cross-protects against human seasonal influenza viruses. Severe infection with avian influenza viruses are known to be associated with delayed immune recovery and impaired lung function (Chen et al., 2016). These consequences may not have increased the susceptibility of the patients who recovered from H7N9 to seasonal influenza infection if subsequent infection occur. It is well known that most people who get influenza will have mild illness and will recover in a few days to less than 2 weeks. However, some people, including young children, adults aged 65 years and older, pregnant women, and people with chronic medical conditions, are among those groups of people who are at higher risk of serious influenza complications (Rothberg et al., 2008). For example, people with chronic lung disease are at higher risk of developing severe pneumonia. Because of severe H7N9 virus infection, the patient had a sequelae of pulmonary interstitial fibrosis although his recovery from the infection. The patient developed severe pneumonia with pH1N1 virus infection, suggesting that a survivor who has recovered from severe H7N9 virus infection may be at a higher risk of having severe complications after subsequent infection with influenza. Therefore, it may be beneficial to include previous H7N9 virus infected patients in the high-risk populations recommended to receive human seasonal influenza vaccination.

## Informed Consent Statement

The study complied with the Declaration of Helsinki and was approved by the Institutional Review Board of Wuxi Center for Disease Control and Prevention. The written informed consent was obtained from the study patient.

## Author Contributions

S-HC, M-NW, Y-HQ, G-LW, G-YM, TZ, and BL conducted the epidemiological investigation, sample, and data collection. M-NW, G-LW, and M-JM performed experiments. M-JM and W-CC conceived and designed the study. M-JM, W-CC, and YY contributed to the writing of the manuscript.

## Conflict of Interest Statement

The authors declare that the research was conducted in the absence of any commercial or financial relationships that could be construed as a potential conflict of interest.
